# Sleep disturbance as a marker of postpartum psychosis risk: a prospective actigraphy study

**DOI:** 10.1186/s12888-025-07017-6

**Published:** 2025-06-02

**Authors:** Chiara Petrosellini, Sofia H. Eriksson, Nicholas Meyer, Edwin Antony, Olivia Protti, Lucinda Donaldson, Vincent van Hees, Aviva Petrie, Andrew McQuillin, Dimitrios Siassakos

**Affiliations:** 1 Elizabeth Garrett Anderson Institute for Women’s Health, University College London, 84-86 Chenies Mews, London, WC13 6HU UK; 2 Division of Psychiatry, University College London, London, UK; 3 Elizabeth Garrett Anderson Wing, University College London Hospitals NHS Foundation Trust, London, UK; 4 Department of Clinical and Experimental Epilepsy, Institute of Neurology, University College London, London, UK; 5 National Hospital for Neurology and Neurosurgery, University College London Hospitals NHS Foundation Trust, London, UK; 6 Insomnia and Behavioural Sleep Medicine Clinic, University College London Hospitals NHS Foundation Trust, London, UK; 7 Department of Psychosis Studies, Institute of Psychology, Psychiatry and Neuroscience, King’s College London, London, UK; 8 East London Mother and Baby Unit, East London NHS Foundation Trust, London, UK; 9 Specialist Perinatal Mental Health Service, North London NHS Foundation Trust, London, UK; 10 Accelting, Almere, The Netherlands; 11 Biostatistics Unit, Eastman Dental Institute, University College London, London, UK

**Keywords:** Postpartum psychosis, Mania, Actigraphy, Sleep disturbance, Sleep quality, Sleep efficiency, Sleep fragmentation, Bipolar disorder

## Abstract

**Background:**

Postpartum Psychosis (PP) is a severe perinatal psychiatric disorder affecting 1–2 in 1000 individuals following childbirth. Most episodes emerge within the first two weeks postpartum and commonly present with mania and decreased need for sleep. The postnatal period is a time of profound sleep disruption and sleep deprivation is a known trigger for mania and psychosis. Despite growing recognition of the role of sleep in the onset and progression of PP, this relationship remains poorly understood. Existing research is largely retrospective, relies on self-reported data and primarily focuses on women with pre-existing bipolar disorder. This prospective study will integrate subjective and objective sleep measures to investigate the relationship between sleep disturbance and postnatal mania. We aim to establish whether sleep patterns in late pregnancy or the early postpartum period can predict mania as a marker of PP.

**Methods:**

This prospective observational cohort study is recruiting pregnant women and will follow participants from the late third trimester until two weeks postpartum. We aim to recruit 100 participants, including individuals with and without psychiatric illness, to ensure broader applicability of the findings and capture the full spectrum of postnatal mania risk. Participants will wear a wrist accelerometer continuously during this period to monitor rest-activity patterns and infer objective sleep parameters including sleep duration, efficiency and fragmentation. Self-reported sleep quality and mood symptoms will be measured using the Pittsburgh Sleep Quality Index (PSQI), Altman Self-Rating Mania Scale (ASRM) and Edinburgh Postnatal Depression Scale (PSQI) at baseline and at days 3–5 and 12–14 postpartum. Actigraphy data will be analysed using the GGIR package in R. Associations between sleep measures and ASRM scores will be assessed using Pearson and Spearman correlation coefficients.

**Discussion:**

This study is the first to prospectively investigate sleep and postnatal mania risk in a cohort including both high- and low-risk individuals. By integrating actigraphy with validated self-report measures, it aims to identify rest-activity patterns that may serve as early indicators of PP. Early recognition of sleep disturbances associated with postnatal mania could inform targeted interventions, improving clinical outcomes for women and families affected by PP.

## Background

Postpartum psychosis (PP) is a severe perinatal mental illness which affects 1–2 in 1000 individuals following childbirth. While delusions and hallucinations are highly prevalent in PP [1], they typically occur in the context of significant affective lability. PP is therefore considered a misnomer [2], as it more closely resembles a severe mood disorder than a primary psychotic illness. Irritability, confusion and anxiety are common features of PP, and episodes of disorientation are often interspersed with periods of lucidity. Due to this varied and rapidly shifting presentation, PP has been described as ‘kaleidoscopic’ [3] and serves as an umbrella term for a spectrum of severe mood and psychotic symptoms following childbirth. Most episodes of PP emerge within the first two weeks postpartum [4], with the majority of women recalling symptom onset by the third day following childbirth [5]. PP typically presents abruptly and escalates rapidly. Thoughts of self-harm are common [1] and delusions are often centred around the baby [6], increasing the risks of suicide and infanticide [7–9]. For these reasons, PP is a psychiatric emergency.

Mania is defined as a period of abnormally and persistently elevated or irritable mood lasting at least one week [10]. Symptoms include inflated self-esteem, reduced need for sleep, pressured speech and impulsive behaviour and these are sufficiently severe to disrupt daily functioning or require hospitalisation. By distorting an individual’s sense of reality, mania can escalate into psychosis and may present with delusions or hallucinations [11]. The lifetime occurrence of a manic episode is the diagnostic hallmark of bipolar disorder (BD), distinguishing it from other mood disorders [12]. In contrast, hypomania is an episodic mood disturbance with symptoms similar to mania but milder in severity, and does not lead to significant social or occupational impairment, hospitalisation or psychosis [10]. Bipolar I disorder is defined by the presence of at least one manic episode, with depression not required for diagnosis, while a diagnosis of bipolar II disorder requires at least one hypomanic and one major depressive episode. It is widely accepted that PP and BD are closely related, and women with pre-existing BD have a significantly increased risk of a manic or psychotic postnatal episode [13]. PP may also be the first presentation of a bipolar illness, and approximately one-third of women who experience first-onset PP will eventually receive a BD diagnosis [14].

Most episodes of PP present with symptoms of mania, among which irritability, decreased need for sleep, pressured speech and elevated mood are particularly common [1, 3, 15]. Women who develop PP frequently report hypomanic symptoms in the early postpartum period [5], and longitudinal studies have shown that subthreshold hypomanic symptoms can predict progression to a BD diagnosis [16, 17]. Additionally, there is evidence that women who experience postnatal hypomanic symptoms have an increased risk of subsequent depression [18–20]. The ability to identify and manage hypomania in the early postpartum period could therefore play a critical role in mitigating subsequent mood disorders. The challenge, however, is that hypomanic symptoms following childbirth are common and are present in at least 10% of all postpartum women [21]. This rate is significantly higher than during pregnancy [22]. While the absence of well-validated, perinatal-specific measurement tools complicates this research, the ability to distinguish true hypomanic symptoms from typical postpartum happiness could offer significant clinical benefits. Not all women with postnatal hypomanic symptoms will develop PP, however all cases represent individuals who are potentially vulnerable to mental health deterioration.

Mania commonly presents with a reduced need for sleep, and sleep deprivation is known to be a potent trigger for both mania [23–26] and psychosis [27–29]. Sleep loss in the early postnatal period is almost universal [30] and aligns with the highest risk window for the onset of PP. Decreased need or ability to sleep is one of the earliest and most common presentations of PP [5], and there is increasing recognition that sleep is likely to play a critical role in its onset and progression. Early studies suggested that sleep deprivation could induce mania in women with PP [31], and longer labours and night time births have indirectly linked peripartum sleep loss to PP onset [32]. More recent research has focused on women with pre-existing BD, who were found to be twice as likely to have had PP if they reported sleep loss as a lifetime trigger for mania [33]. A subsequent prospective study showed a five-fold increased risk of PP in women with BD who experienced intrapartum sleep loss compared to those who did not, with all episodes occurring within three weeks postpartum [34]. Despite growing evidence linking sleep disruption to PP, much remains to be understood about the nature of this relationship. It is unclear whether sleep disturbance serves as a trigger for PP, a prodromal symptom or both, and the underlying neurobiological and psychosocial mechanisms are complex and poorly understood. Existing research is largely retrospective, relies entirely on self-reported data and has focused predominantly on women with BD, leaving uncertainty about its applicability to the general population. To address these limitations, we are conducting a prospective study that integrates subjective with objective measures of sleep and is not limited to women with BD, aiming to capture the full spectrum of postpartum mania risk.

Actigraphy is a non-invasive method that uses accelerometry sensors to record movement in order to infer physical activity, sleep-wake patterns and circadian rhythms over time. This technique offers a cost-effective, non-invasive alternative to polysomnography for objective, longitudinal assessment of sleep in both research and clinical settings [35]. Actigraphy can be conducted at various body sites, such as the ankle, leg or waist, however the use of a wrist-accelerometer on the non-dominant hand is most common [36]. This tool offers a valuable method for monitoring sleep patterns during the perinatal period, particularly for postpartum mothers. Traditional sleep assessment methods such as polysomnography are intrusive and limited to short recording periods, making them poorly suited to capturing the irregular sleep patterns which are common among new parents. Actigraphy provides an objective estimate of sleep with minimal disruption, making it well-suited to this population [37]. Additionally, the widespread adoption of wearable devices with accelerometry sensors has familiarised the public with this technology [36], enhancing its acceptability and supporting the translation of research into clinical practice. In this study, we use ‘accelerometer’ to refer to the wearable device and ‘actigraphy’ to describe the method of analysing the movement data it generates, though both terms refer to the same underlying technology.

Actigraphy has increasingly been used to study sleep and circadian disruptions in BD and psychosis. Individuals with psychotic disorders often demonstrate significant rest-activity abnormalities that can be tracked with actigraphy, providing valuable insights into mood instability and impulsivity [38–40]. In BD, actigraphy can capture the dynamic interactions between motor activity, energy, mood and sleep [41, 42] and has been shown to predict deterioration in mental state [43]. Reductions in sleep duration [28] and sleep efficiency [44] tracked by actigraphy have also been associated with the worsening or emergence of psychosis, creating opportunities for real-time monitoring and targeted interventions that could improve outcomes.

This study aims to combine actigraphy with subjective sleep measures to identify rest-activity patterns associated with postnatal mania, providing an early indicator of PP risk. As the symptoms of PP most commonly arise within the first two weeks postpartum, our study will focus on this critical window. This work is driven by the following hypotheses:

(i) Sleep disturbance precedes the emergence of manic symptoms associated with PP.
(ii) Screening for sleep changes in late pregnancy and/or the early postnatal period can help identify individuals who are at increased risk of postnatal (hypo)mania.

We aim to establish whether subjective and/or objective measures of sleep during the late third trimester and the early postpartum period can be used to predict symptoms of mania. Early detection through this approach could enable timely interventions, helping to prevent the potentially devastating outcomes associated with PP.

## Methods

### Study design and setting

This is a prospective, observational cohort study which is currently recruiting participants at University College London Hospital (UCLH) in the United Kingdom. UCLH provides acute and specialist services in six hospitals in central London, including the Elizabeth Garrett Anderson (EGA) Wing and the National Hospital for Neurology and Neurosurgery (NHNN). The EGA Wing at UCLH is a specialist maternity unit which manages approximately 5500 births each year. It provides comprehensive antenatal, intrapartum and postnatal care, including tertiary-level maternal and fetal medicine services. The NHNN is the largest specialist centre for neurological and neurosurgical care in the United Kingdom. Its sleep neurology service offers pioneering expertise in the diagnosis and treatment of neurological sleep disorders, conducting hundreds of actigraphy and inpatient polysomnography studies annually. In addition, the insomnia and behavioural sleep medicine clinic at UCLH provides multidisciplinary assessment and treatment for insomnia and sleep-related disorders. This project is a collaboration between all of these specialist services, with support from local community perinatal mental health services. Study participants are enrolled in the study during the late third trimester of pregnancy and are followed-up until two weeks postpartum.

### Participants

Potential participants are identified through midwifery-led and high-risk antenatal clinics at UCLH. Recruitment posters have also been displayed in antenatal areas throughout the hospital and provide contact details for potential participants to make direct contact with the study team. Women are eligible for the study irrespective of whether or not they have a history of mental illness and are recruited according to the following criteria (Table 1):

**Table 1:** Study eligibility criteria

INCLUSION CRITERIA	EXCLUSION CRITERIA
• ≥ 18 years of age • Pregnant and ≥ 37 weeks’ gestation • Able and willing to wear a wristwatch for 2–6 weeks	• Previous skin irritation or hypersensitivity to silicone • Intrauterine fetal death • In active labour at the time of recruitment • Inability to provide written informed consent

### Clinical data and screening tools

At the time of enrolment into the study, baseline clinical data is collected for each study participant including general medical, obstetric and psychiatric history, which are recorded in a bespoke case report form. Participants are also asked to complete Pittsburgh Sleep Quality Index (PSQI), Altman Self-Rating Mania Scale (ASRM) and Edinburgh Postnatal Depression Scale (EPDS) assessments.

#### Pittsburgh sleep quality index (PSQI)

The PSQI is a self-assessment tool designed to assess sleep quality and disturbances over a one-month time period [45]. Its items are used to generate 7 component scores: subjective sleep quality, sleep latency, sleep duration, sleep efficiency, sleep disturbances, use of sleep medication and daytime dysfunction. Each component is scored on a scale from 0 to 3 and combined to produce a global score that reflects overall sleep quality (range 0 to 21), with higher scores indicating poorer sleep quality. The PSQI is one of the most rigorously validated tools used in sleep diagnostics and has been used extensively in perinatal cohorts [30]. A global PSQI score ≥ 5 is widely used as the threshold for identifying individuals with poor sleep quality [45], though it has been proposed that a higher cut-off may be more appropriate in perinatal populations to better differentiate transient sleep changes from clinically significant disturbance [46].

#### Altman self-rating mania scale (ASRM)

The ASRM is a self-assessment questionnaire designed to assess the presence and severity of manic symptoms over a one-week period [47]. It consists of 5 items that assess key features of mania: elevated mood, increased self-confidence, decreased need for sleep, increased talkativeness or pressured speech and increased motor activity or energy. Each item is scored on a scale from 0 to 4, with higher scores indicating more severe manic symptoms. The total score ranges from 0 to 20 and a cut-off score of 6 is used to identify individuals with a high probability of a manic or hypomanic episode and who require further assessment. The ASRM has been validated for use in individuals with bipolar disorder, depression, schizophrenia and schizoaffective disorder and has been applied to perinatal cohorts in the literature [48, 49].

In individuals with BD, ASRM scores have been shown to increase the probability of detecting an abrupt change in mental state [50]. Scores on the ASRM have been shown to correlate well with scores on clinician administered scores such as the Clinician-Administered Rating Scale for Mania (CARS-M) and the Young Mania Rating Scale (YMRS) [51], and with scores on The Highs Scale, a screening tool designed to detect hypomania in the postpartum period [18, 48]. While the Highs Scale was specifically developed and validated for the perinatal population, the ASRM was chosen for this study because it focuses on core manic symptoms as defined by the Diagnostic and Statistical Manual of Mental Disorders (DSM) [10] and has been widely validated in individuals with bipolar disorder, a key risk factor for PP. Unlike the Highs Scale, which captures a broader range of high-energy states, the ASRM is therefore more likely to distinguish pathological mania from typical postpartum experiences. Additionally, its sensitivity to abrupt mood changes enhances its clinical utility in this population.

#### Edinburgh postnatal depression scale (EPDS)

The EPDS is a self-report questionnaire used to screen for symptoms of depression in the perinatal period [52]. It is validated for use both antenatally and postnatally [53, 54]. It consists of 10 questions that assess common symptoms of depression over a one-week period. Each item is scored on a scale from 0 to 3, with a total possible score ranging from 0 to 30. A cut-off score of 13 is used to identify individuals who may need further assessment or intervention, as the likelihood of depression is high. Whilst this study does not specifically aim to investigate perinatal depression, the inclusion of regular EPDS assessments is necessary to account for depression as a potential confounder associated with sleep disturbance.

All study participants are invited to repeat PSQI, ASRM and EPDS assessments once within each of the following timeframes:

- Day 3–5 postpartum.
- Day 12–14 postpartum.

These time points were selected to capture early symptom onset within the high-risk period for PP and to ensure comparability with previous review studies of postpartum mania [21, 55]. At these postpartum time points, participants are instructed to complete all questionnaires, including the PSQI, based on their experiences in the last week. While the PSQI is designed to assess sleep over a one month period, this adjustment was made to minimise the influence of antenatal sleep patterns on responses and to capture acute postpartum sleep changes more accurately.

At the end of the study period, participants’ maternity records are screened to identify perinatal complications which may act as confounders. These are systematically recorded in a bespoke perinatal report form for each participant. Any clinical concerns during study follow-up are escalated according to the study’s escalation protocol for mental distress and safeguarding. Participation in the study does not influence the delivery of routine clinical care, and if any deterioration in mental state is detected during study procedures, the relevant clinical teams (including 24 h mental health crisis teams) will be notified immediately.

### Wrist actigraphy

Study participants are asked to wear a wrist accelerometer continuously from the time of recruitment in the late third trimester until the time of completion of the final screening questionnaires on day 12–14 postpartum. This will record rest-activity patterns to estimate objective sleep variables including sleep duration, sleep efficiency and sleep fragmentation. The device chosen for the study is MotionWatch8 by CamNtech [56]. This is a medical-grade actigraphy watch designed for the monitoring of sleep. It has a proven track record for data quality and reliability in large-scale clinical trials [57–60] and is the device currently used for outpatient sleep studies by the sleep neurology service at NHNN. The device is set to a 30-second epoch length for data interpretation, with each epoch representing a 30-second interval for which the recorded activity is summarised. Participants are encouraged to press the button on the device to mark the start and end of each sleep period, helping to align self-reported sleep times with the actigraphy data for more accurate analysis. Sleep diaries were omitted to minimise participant burden, based on evidence that accelerometer data can reliably estimate key sleep parameters without them [61], including in the perinatal period [62].

**Fig. 1:**
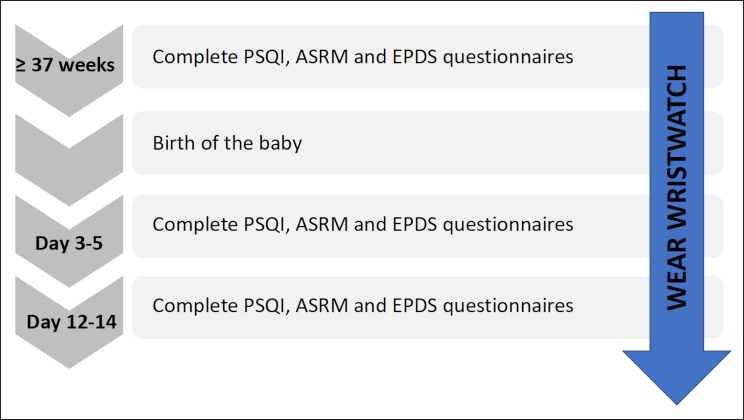
Study flow diagram. PSQI = Pittsburgh Sleep Quality Index; ASRM = Altman Self-Rating Mania scale; EPDS = Edinburgh Postnatal Depression Scale

An overview of the study flow is shown in Fig. 1.

### Ethical approval and research governance

The Sleep, cLock gEnes and Education in Postpartum Psychosis (SLEEPP) Study was approved by the London - City & East Research Ethics Committee (REC) on 17/01/2024 (reference: 23/LO/0960). The study was registered with the UCL Data Protection Office (reference: Z6364106/2023/06/85) and all data were processed in accordance with the General Data protection Regulation requirements. Confirmation of capacity and capability to begin this study at UCLH was received on 16/02/2024 and recruitment is ongoing.

### Patient and public involvement

Close liaison with the Action on Postpartum Psychosis (APP) charity [63] has been integral to the development of this study. APP is the largest national network offering peer support for women and families affected by PP. They promote public awareness of PP on international platforms, run educational workshops and facilitate research in PP. We have continued to work with APP throughout the implementation of this project to ensure that our objectives remain in line with the needs of people who access their support services. Through APP, we have created a key stakeholder group which includes several individuals with lived experience of PP. The study protocol and all participant-facing materials were designed in partnership with this group.

### Data analysis

The actigraphy data stored in the MotionWatch8 devices is downloaded for further analysis in R [64]. This provides a summary of activity and light exposure for each 30-second epoch, enabling detailed quantitative analysis of the sleep and wake behaviours across the monitoring period. The GGIR open-source package [65] will be used to process the data and compute a range of sleep, physical activity and circadian rhythm parameters. GGIR is one of the most widely used tools for analysing accelerometer data in health research and has been extensively adopted for its robustness and reproducibility. Whilst GGIR was primarily developed to process raw accelerometer data expressed in gravitational units, it also offers functionalities to process epoch-level accelerometer data.

All actigraphy variables will be summarised for each participant overall and for each of three study time intervals:

1. From the time of recruitment in pregnancy until childbirth.
2. From the time of birth until the time of completion of the ASRM on day 3–5.
3. From the time of completion of the ASRM on day 3–5 to the time of completion of the ASRM on day 12–14.

**Fig. 2:**
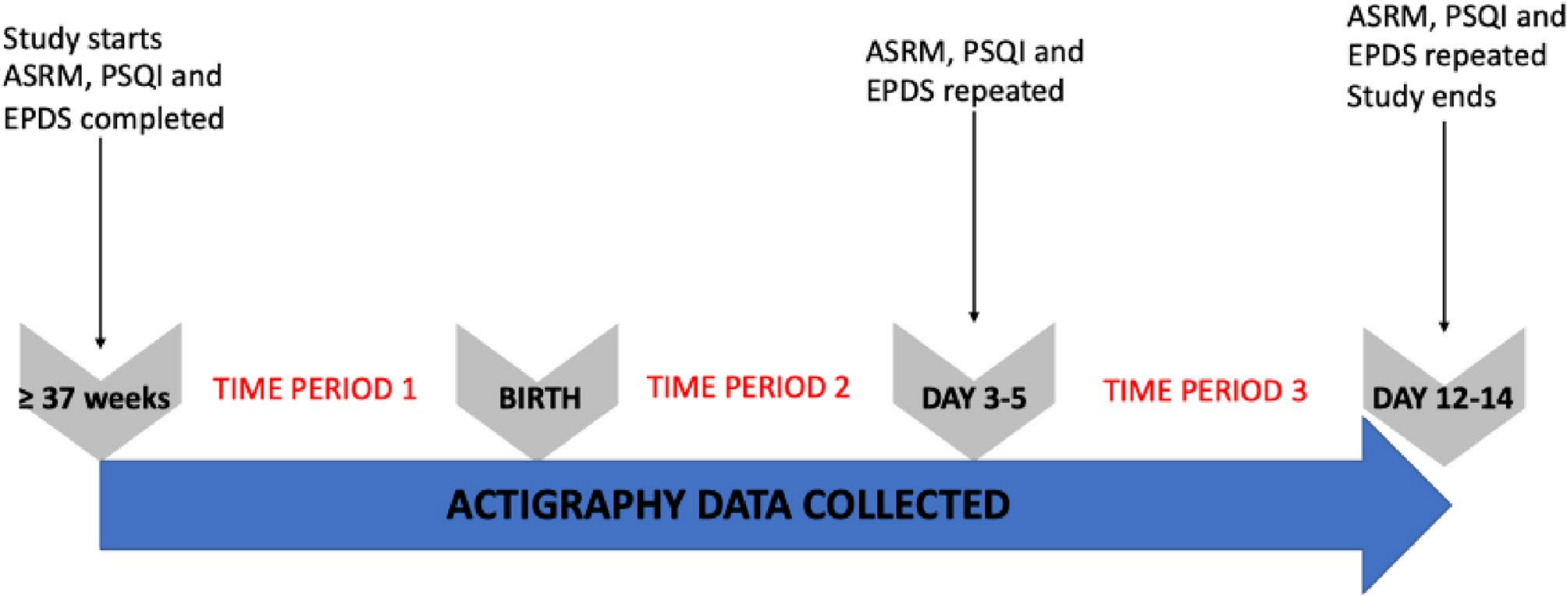
Time intervals used for data analysis. PSQI = Pittsburgh Sleep Quality Index; ASRM = Altman Self-Rating Mania scale; EPDS = Edinburgh Postnatal Depression Scale

These time intervals are illustrated in the diagram below (Fig. 2).

Using the data collected, scatter diagrams will be used to visualise:

- PSQI scores plotted against ASRM scores for each participant at each time point.
- Actigraphy-derived variables plotted against ASRM scores for each participant at each time point.

Pearson or Spearman correlation coefficients, as appropriate, will be estimated and hypothesis tests will be conducted to determine if each differs significantly from zero at each time point. Correlations will also be examined across time points, to assess whether sleep variables measured in pregnancy or at the first postnatal assessment are associated with ASRM scores measured later in the study. Where sample size permits, logistic regression analysis will be used to test whether PSQI scores and/or actigraphy variables are predictive of a positive mania screen (ASRM score ≥ 6) at each time point and to test whether PSQI scores and/or actigraphy variables at a given time point are predictive of a positive mania screen at a future time point. Depressive symptoms (captured via the EPDS scores) and perinatal complications which could act as confounders will be adjusted for where possible.

### Sample size

An accurate power calculation for the determination of the optimal sample size for the Pearson correlation coefficient requires an indication of the correlation coefficient (r) between the variables being assessed [66]. The expected correlation coefficient between sleep variables and ASRM scores, however, is unknown. We aim to identify associations of *r* ≥ 0.3, reflecting a small to moderate effect size which could be clinically meaningful in this context. Assuming a two-sided test with α = 0.05, power (1 − β) = 0.80, and an assumed correlation coefficient of *r* = 0.3, a minimum sample size of 84 participants would be required to detect a statistically significant association [67]. We aim to recruit 100 participants to allow for potential missing data and to ensure adequate representation across the perinatal population.

## Discussion

To the best of our knowledge, this is the first prospective study to examine the relationship between sleep variables and postnatal mania risk. By integrating actigraphy parameters with validated self-report measures, this study aims to identify rest-activity patterns that may serve as early indicators of postpartum psychosis (PP). While PP is a serious perinatal mental illness, outcomes for most women are excellent when treatment is commenced promptly [68, 69]. With early intervention, most women with first-onset PP will not experience further episodes of severe mental illness [14] and will have good functional recovery [70]. Effective treatment is dependent on the ability to predict, recognise and escalate the early signs.

Early identification of sleep-related risk offers an opportunity for greater collaborative working between obstetric and psychiatric services. Incorporating sleep science into perinatal care has the potential to refine screening for mental illness, improve the delivery of preventative support services and inform the design of maternity pathways. This could include adjustments to birth planning, induction of labour protocols, infant feeding support and postnatal ward arrangements to minimise sleep disruption. Multidisciplinary early intervention may also obviate the need for psychiatric hospital admission, which can be a traumatic experience and may involve separation from the infant in cases where mother and baby unit care is unavailable or unsuitable [71]. This has important implications for mother-infant bonding and attachment.

This study has several methodological strengths. Its prospective design minimises recall bias by collecting data in real time, improving reliability of any associations observed between sleep patterns and mania risk. The use of actigraphy provides objective rest-activity estimates, overcoming limitations associated with self-reported sleep data. Furthermore, inclusion of participants with and without pre-existing psychiatric illness broadens the study’s applicability and enhances its potential impact on postnatal mental health management. Assessing participants at multiple time points within the critical window for PP allows for a dynamic understanding of symptom evolution, offering a nuanced perspective on sleep disturbances as potential early indicators. Additionally, the use of GGIR ensures standardised and reproducible data processing for sleep, activity and circadian parameters.

When drawing conclusions on any associations between sleep data and mania risk, it is important to acknowledge the challenges in distinguishing pathological mood elevation from typical postpartum experiences. The ASRM cut-off has been criticised for likely overestimating hypomania in the postpartum period [48], as it may capture transient high-energy states that do not necessarily indicate mania. Nevertheless, if this study establishes a significant relationship between a specific, quantifiable sleep parameter and ASRM scores, this association can be extrapolated to infer the sleep changes that might indicate a higher risk of clinically significant mania. Modelling this relationship could provide a framework for using key sleep parameters to identify individuals at highest risk. An additional challenge is that some women with PP may present with a mixed affective state, in which manic and depressive symptoms occur simultaneously [72]. Analysing the relationship between EPDS and ASRM scores in this cohort may provide further insight into the complexity of mixed affective episodes in the early postpartum period.

It is also essential to consider the limitations of actigraphy, as accelerometers indirectly infer sleep from movement patterns. Actigraphy demonstrates high sensitivity in detecting sleep compared to polysomnography, but lower specificity in identifying wakefulness, which may lead to an overestimation of total sleep time [36]. Traditional actigraphy devices do not reliably measure sleep architecture [73] and will not account for external influences on maternal activity, such as infant sleep patterns. Despite these challenges, wearable devices are rapidly improving in accuracy and usability, offering a promising avenue for monitoring sleep patterns in perinatal populations. Their ability to provide continuous, objective sleep data in real-world settings makes them particularly valuable for monitoring dynamic changes in risk during the perinatal period.

In summary, this study represents a significant step in understanding the complex relationship between sleep and PP. Early recognition of sleep and rest-activity patterns associated with mania could play a pivotal role in improving timely intervention in postpartum mood disorders. This approach has the potential to inform future strategies for prevention and treatment, leading to improved outcomes for women and families affected by PP.

## Data Availability

No datasets were generated or analysed during the current study.

## Abbreviations

ASRM: Altman Self-Rating Mania Scale
APP: Action on Postpartum Psychosis
DSM: Diagnostic and Statistical Manual of Mental Disorders
EDPS: Edinburgh Postnatal Depression Scale
EGA: Elizabeth Garrett Anderson
NHNN: National Hospital for Neurology and Neurosurgery
PP: Postpartum Psychosis
PSQI: Pittsburgh Sleep Quality Index
REC: Research Ethics Committee
SLEEPP: Sleep, cLock gEnes and Education in Postpartum Psychosis
UCL: University College London
UCLH: University College London Hospital
